# Dr. Kirtley Ryland

**Published:** 1855-05

**Authors:** 


**Dr, Kirtly Ryland.**

This gentleman has permanently located in Moline, Rock Island County, Illinois, where we hope his talents and fine acquirements will be properly appreciated by the citizens of that thriving town and of the surrounding country.

Dr. R. is a young man of as much scientific promise as any young gentleman with whom we have the pleasure of an acquaintance. During his stay with us he exhibited talents of a superior order, and acquirements thorough and extensive. He instructed the class of the College in practical Anatomy during last winter, besides delivering a series of lectures on Medical Jurisprudence to the satisfaction of the Faculty, at whose request the task was undertaken, and also to the edification and gratification of the class. He is a fine writer, several of his communications having appeared in this Journal, and copied extensively in our eastern and southern medical periodicals.

We ask of the profession in that region, that attention, respect and confidence in his favor, which a learned and accomplished young physician merits and should receive, when he launches his bark in strange and unknown waters. He has our best wishes for his success.

